# Clinical Relevance and Discriminatory Value of Elevated Liver Aminotransferase Levels for Dengue Severity

**DOI:** 10.1371/journal.pntd.0001676

**Published:** 2012-06-05

**Authors:** Linda K. Lee, Victor C. Gan, Vernon J. Lee, Adriana S. Tan, Yee Sin Leo, David C. Lye

**Affiliations:** 1 Department of Clinical Epidemiology, Tan Tock Seng Hospital, Singapore, Singapore; 2 Department of Infectious Diseases, Tan Tock Seng Hospital, Singapore, Singapore; 3 Department of Epidemiology and Public Health, National University of Singapore, Singapore, Singapore; 4 Department of Medicine, Yong Loo Lin School of Medicine, National University of Singapore, Singapore, Singapore; Pediatric Dengue Vaccine Initiative, United States of America

## Abstract

**Background:**

Elevation of aspartate aminotransferase (AST) and alanine aminotransferase (ALT) is prominent in acute dengue illness. The World Health Organization (WHO) 2009 dengue guidelines defined AST or ALT≥1000 units/liter (U/L) as a criterion for severe dengue. We aimed to assess the clinical relevance and discriminatory value of AST or ALT for dengue hemorrhagic fever (DHF) and severe dengue.

**Methodology/Principal Findings:**

We retrospectively studied and classified polymerase chain reaction positive dengue patients from 2006 to 2008 treated at Tan Tock Seng Hospital, Singapore according to WHO 1997 and 2009 criteria for dengue severity. Of 690 dengue patients, 31% had DHF and 24% severe dengue. Elevated AST and ALT occurred in 86% and 46%, respectively. Seven had AST or ALT≥1000 U/L. None had acute liver failure but one patient died. Median AST and ALT values were significantly higher with increasing dengue severity by both WHO 1997 and 2009 criteria. However, they were poorly discriminatory between non-severe and severe dengue (e.g., AST area under the receiver operating characteristic [ROC] curve = 0.62; 95% confidence interval [CI]: 0.57–0.67) and between dengue fever (DF) and DHF (AST area under the ROC curve = 0.56; 95% CI: 0.52–0.61). There was significant overlap in AST and ALT values among patients with dengue with or without warning signs and severe dengue, and between those with DF and DHF.

**Conclusions:**

Although aminotransferase levels increased in conjunction with dengue severity, AST or ALT values did not discriminate between DF and DHF or non-severe and severe dengue.

## Author Summary

Dengue is a global public health problem, as the incidence of the disease has reached hyperendemic proportions in recent decades. Infection with dengue can cause acute, febrile illness or severe disease, which can lead to plasma leakage, bleeding, and organ impairment. One of the most prominent clinical characteristics of dengue patients is increased aspartate and alanine aminotransferase liver enzyme levels. The significance of this is uncertain, as it is transient in the majority of cases, and most patients recover uneventfully without liver damage. In this study, we characterized this phenomenon in the context of dengue severity and found that, although liver enzyme levels increased concurrently with dengue severity, they could not sufficiently discriminate between dengue fever and dengue hemorrhagic fever or between non-severe and severe dengue. Therefore clinicians may need to use other parameters to distinguish dengue severity in patients during early illness.

## Introduction

Dengue is a mosquito-borne arboviral infection endemic to most tropical and subtropical countries [1]. Elevation of the liver enzymes aspartate aminotransferase (AST) and alanine aminotransferase (ALT) is common in acute dengue illness, occurring in 65–97% [2], [3], [4], [5] of dengue patients, peaking during the convalescent period of illness (days 7–10) [2], [4], [6]. In dengue-endemic countries, dengue is an important cause of acute viral hepatitis [7].

Elevated AST and ALT levels have been associated with bleeding [2], [4], [6] and dengue hemorrhagic fever (DHF) [3], [8]. Liver failure has been recognized as a complication and unusual manifestation of dengue [9], [10] but occurred infrequently in 3 of 270 patients in Taiwan [6] and 5 of 644 patients in Vietnam [4]. In Malaysia, 8 of 20 pediatric DHF patients developed liver failure, 1 died, and the rest recovered completely [11]. In Singapore, AST or ALT levels were not independent predictors of DHF in 1973 adult dengue patients [12].

In 2009, the World Health Organization (WHO) revised its dengue guidelines and proposed severe organ impairment as one category of severe dengue in addition to severe plasma leakage and severe bleeding [1]. Severe liver involvement was defined as AST or ALT≥1000 units/liter (U/L). In Taiwan, AST>10 times the upper limit of normal (ULN) occurred in 11% of dengue patients [6], while in Brazil this occurred in 4% of their cohort [3]. In this study, we aimed to evaluate the clinical relevance of elevated AST and ALT levels and correlate liver aminotransferase levels with dengue severity according to WHO 1997 and 2009 classifications.

## Methods

All laboratory-confirmed dengue patients identified from our hospital microbiology database and treated using a standardized dengue clinical care path at the Department of Infectious Diseases, Tan Tock Seng Hospital (TTSH), Singapore from 2006 to 2008 were retrospectively reviewed for demographic, serial clinical and laboratory, radiological, treatment, and outcome data. These cases were positive by real-time polymerase chain reaction (PCR) [13]. We included patients with only positive dengue serology in only subgroup analyses, as we did not have paired sera, and other etiologies for elevated AST and ALT could not be excluded without more extensive evaluation.

Cases were categorized using serial clinical and laboratory data from the entire clinical course as dengue fever (DF), DHF, or dengue shock syndrome (DSS) using WHO 1997 classifications [9]. Dengue fever classification requires fever and at least two of the following: headache, eye pain, myalgia, arthralgia, rash, bleeding, and leukopenia. Dengue hemorrhagic fever requires all of the following: fever, platelet count ≤100×10^9^/liter, bleeding, and plasma leakage [9]. Dengue shock syndrome is a case of DHF with either tachycardia and pulse pressure <20 mmHg or systolic blood pressure <90 mmHg [9].

Cases were also categorized as dengue without warning signs (WS), dengue with WS, or severe dengue using WHO 2009 classifications [1]. Dengue (WHO 2009) requires fever and two of the following: nausea, vomiting, rash, aches and pains, leukopenia, or any warning sign [1]. Warning signs include abdominal pain or tenderness, persistent vomiting, clinical fluid accumulation, mucosal bleeding, lethargy or restlessness, hepatomegaly, or hematocrit rise (≥20%) with rapid drop in platelet count (<50,000/liter) [1], [14]. We modified the WHO 2009 warning sign of rise in hematocrit concurrent with rapid drop in platelet count by quantifying it as hematocrit ≥20% concurrent with platelet count <50,000/liter, as this was shown to correlate significantly with dengue death in our adult dengue death study [14]. Severe dengue includes severe plasma leakage, severe bleeding, and severe organ impairment [1].

We performed a subgroup analysis for median maximum AST and ALT values stratified by febrile (days 1–3 of illness), critical (days 4–6), and convalescent (days 7–10) phases as defined by WHO 2009 [1] and compared across dengue severity classification according to WHO 1997 [9] and 2009 [1].

We excluded severe dengue due to isolated elevation of AST or ALT≥1000 U/L from our definition of severe dengue outcome, as this would be a confounder in assessing the relevance of AST or ALT levels in defining dengue severity. Patients had AST/ALT taken at presentation and then throughout hospitalization at the physician's discretion. Maximum AST and ALT values recorded at a median of 4 days of illness (interquartile range [IQR]: 3–5 days) were used in this analysis. Those with pre-existing liver diseases were excluded. At TTSH, the ULN for AST is 41 U/L; for ALT, it is 63 U/L for males and 54 U/L for females.

We assessed the clinical relevance of elevated AST or ALT levels using four liver failure criteria—two for acute liver failure, and two that determine prognosis from chronic liver disease. The American Association for the Study of Liver Diseases (AASLD) recommends defining acute liver failure in a patient as: international normalized ratio (INR)≥1.5, any degree of altered mental status, and illness <26 weeks in duration without pre-existing cirrhosis [15]. The King's College criteria assess prognoses in those with acute liver failure; the criteria are: prothrombin time >100 seconds or 3 of the following: age >40 years, prothrombin time >50 seconds, serum bilirubin >18 mg/dL, time from jaundice to encephalopathy >7 days [16]. The model for end-stage liver disease (MELD) determines three-month mortality based on the following formula: 3.8×(log serum bilirubin [mg/dL])+11.2×(log INR)+9.6×(log serum creatinine [mg/dL])+6.4 [17]. The Child-Pugh criteria include assessment of degree of ascites, serum bilirubin and albumin, prothrombin time, and encephalopathy to determine one- and two-year survival [18].

The Mann-Whitney U and Kruskal-Wallis tests were used to determine statistical significance for continuous variables, and chi-square or Fisher's exact test for categorical variables. Statistical tests were conducted at the 5% level of significance. Receiver operating characteristic (ROC) curves showing the area under the curve (AUC) were generated to determine the discriminatory performance of aminotransferase values. All statistical analyses were performed using Stata 10 (Stata Corp., College Station, TX).

This was a retrospective study involving data collection from medical records. All patient data were anonymized during analysis. This study was approved by the Institutional Review Board, National Healthcare Group, Singapore [DSRB E/08/567].

## Results

From 2006 to 2008, 690 dengue PCR positive cases were reviewed. Males comprised 493 (71%) of the cases, and the median age of the cohort was 35 years (IQR: 27–43 years). A Charlson comorbidity index ≥3, which predicts increased one-year mortality [19], was noted in 5 (0.7%) patients. With WHO 1997 classification, 62% had DF, 31% DHF, and 7% DSS. With WHO 2009 classification, 14% had dengue, 62% had dengue with warning signs, and 24% had severe dengue. Hence, by WHO 1997 classification, 38% of patients with DHF/DSS needed close monitoring, while by WHO 2009 classification, 86% of patients with warning signs or severe dengue required close monitoring.

Median length of illness from onset to hospital presentation was 4 days (IQR: 3–5 days), while median length of hospital stay was 5 days (IQR: 4–6 days). Intravenous fluid was administered to 641 (93%) and platelet transfusion to 86 (12%). Intensive care unit (ICU) admission was required in 3 patients, and death occurred in 1 patient due to pneumonia.

### (1) Elevation of AST/ALT and risk of liver failure

Overall, 595 (86%) had AST above the ULN, and 316 (46%) had ALT above the ULN. Seven patients (1.0%) had severe dengue according to WHO 2009 criteria concurrent with AST or ALT≥1000 U/L while three additional patients had severe dengue due to AST or ALT≥1000 U/L only. Of the former seven patients, 86% had severe plasma leakage, 29% had severe bleeding, and none had severe organ impairment other than isolated AST or ALT≥1000 U/L. Among the 3 patients admitted to the ICU, AST or ALT values were above the ULN but below 1000 U/L.

No patients in our cohort developed acute liver failure under AASLD or King's College criteria. With Child-Pugh scoring, 2 (0.3%) belonged to Child-Pugh class C. With MELD scoring, predicted three-month mortality of 6% were identified in 68 (10%) patients in our cohort and 19.6% in 2 (0.3%) patients. The same two patients who were Child-Pugh class C also had a predicted 19.6% three-month mortality using MELD scoring; they both had DSS and severe dengue.

### (2) Dengue severity and AST/ALT values

Median AST and ALT values for dengue without warning signs, dengue with warning signs, and severe dengue (Table 1) were 83.5 U/L, 92 U/L, and 124 U/L, respectively (*p*<0.001); median ALT values were 49 U/L, 53 U/L, and 73.5 U/L (*p* = 0.002). Table 2 shows median AST and ALT values for patients with DF, DHF, and DSS. Median AST values for these categories were 93 U/L, 103 U/L, and 137.5 U/L, respectively (*p* = 0.01), and median ALT values were 52 U/L, 60 U/L, and 74 U/L (*p* = 0.05).

**Table 1:** AST and ALT distributions by WHO 2009 dengue classification.

	Dengue without warning signs (n = 100)	Dengue with warning signs (n = 426)	Severe dengue (n = 164)	*P* value
AST, U/L	83.5 (48.5–153.5)	92 (57–167)	124 (75–244.5)	<0.001
ALT, U/L	49 (28–113)	53 (32–100)	73.5 (40–147.5)	0.002

AST = aspartate aminotransferase.

ALT = alanine aminotransferase.

U/L = units/liter.

All values are expressed as median (interquartile range).

**Table 2:** AST and ALT distributions by WHO 1997 dengue classification.

	Dengue fever (n = 429)	Dengue hemorrhagic fever (n = 211)	Dengue shock syndrome (n = 50)	*P* value
AST, U/L	93 (55–165)	103 (66–203)	137.5 (63–265)	0.01
ALT, U/L	52 (31–107)	60 (37–118)	74 (35–167)	0.05

AST = aspartate aminotransferase.

ALT = alanine aminotransferase.

U/L = units/liter.

All values are expressed as median (interquartile range).

In a separate analysis of our serology-positive cohort (n = 1487), median AST values for dengue without WS, dengue with WS, and severe dengue were 84 U/L, 114 U/L, and 147 U/L (*p*<0.001). Median ALT values were 56 U/L, 73 U/L, and 97.5 U/L (*p* = 0.01). For patients with DF, DHF, and DSS, median AST values were 105 U/L, 130 U/L, and 129 U/L (*p*<0.001), and median ALT values were 68 U/L, 78 U/L, and 85.5 U/L (*p* = 0.008).

In other hemorrhagic fevers, higher AST∶ALT ratios correlated with disease fatality [20]. In our PCR-positive cohort, median AST∶ALT ratios for DF, DHF, and DSS were 1.68, 1.68, and 1.88 (*p* = 0.29) and for dengue without WS, dengue with WS, and severe dengue, they were 1.60, 1.68, and 1.78 (*p* = 0.10), respectively.

### (3) Aminotransferase levels stratified by febrile, critical and convalescent phases

The majority of our patients' maximum AST and ALT values were recorded during febrile (n = 258) and critical (n = 377) phases of acute dengue illness. By WHO 2009 dengue severity classification, the median AST and ALT values were significantly higher for severe dengue compared to dengue with and without warning signs during both the febrile and critical phases but not the convalescent phase (Table 3). By WHO 1997 classification, the median AST and ALT values were significantly higher for DHF versus DF and DSS in the febrile phase only but not critical and convalescent phases (Table 4).

**Table 3:** AST and ALT distributions by dengue phase and WHO 2009 classification.

	Phase of illness	Dengue	Dengue with warning signs	Severe dengue	*P* value
AST, U/L	Febrile phase (days 1–3)	56.5 (39–107)	68 (48–103)	106 (57.5–167)	0.004
	Critical phase (days 4–6)	93 (61–158)	108.5 (64–189.5)	143.5 (82–265)	0.004
	Convalescent phase (days 7–10)	196 (112–227)	278 (171–413)	255 (137–439)	0.56
ALT, U/L	Febrile phase (days 1–3)	31.5 (22–50)	45 (24–65)	56 (27.5–107.5)	0.02
	Critical phase (days 4–6)	54.5 (34.5–118.5)	60.5 (34.5–105)	72 (40–165)	0.05
	Convalescent phase (days 7–10)	130 (60–223)	215.5 (150.5–375)	131 (97–301)	0.22

AST = aspartate aminotransferase.

ALT = alanine aminotransferase.

U/L = units/liter.

All values are expressed as median (interquartile range).

**Table 4:** AST and ALT distributions by dengue phase and WHO 1997 classification.

	Phase of illness	Dengue fever	Dengue hemorrhagic fever	Dengue shock syndrome	*P* value
AST, U/L	Febrile phase (days 1–3)	63 (41–111.5)	84.5 (59.5–139)	60 (41–107)	0.02
	Critical phase (days 4–6)	101.5 (67–190.5)	123 (66–211)	163.5 (86–285.5)	0.12
	Convalescent phase (days 7–10)	257.5 (140–296)	286 (188–413)	229 (137–476)	0.65
ALT, U/L	Febrile phase (days 1–3)	39.5 (24–65)	50.5 (34–80)	31.5 (19–67)	0.03
	Critical phase (days 4–6)	58 (36–111)	62.5 (35–120)	69.5 (42–146.5)	0.57
	Convalescent phase (days 7–10)	171 (108–303)	212 (130–334)	301 (104–473)	0.44

AST = aspartate aminotransferase.

ALT = alanine aminotransferase.

U/L = units/liter.

All values are expressed as median (interquartile range).

### (4) Does a threshold AST or ALT value defining severe dengue exist?

**Figure 1:**
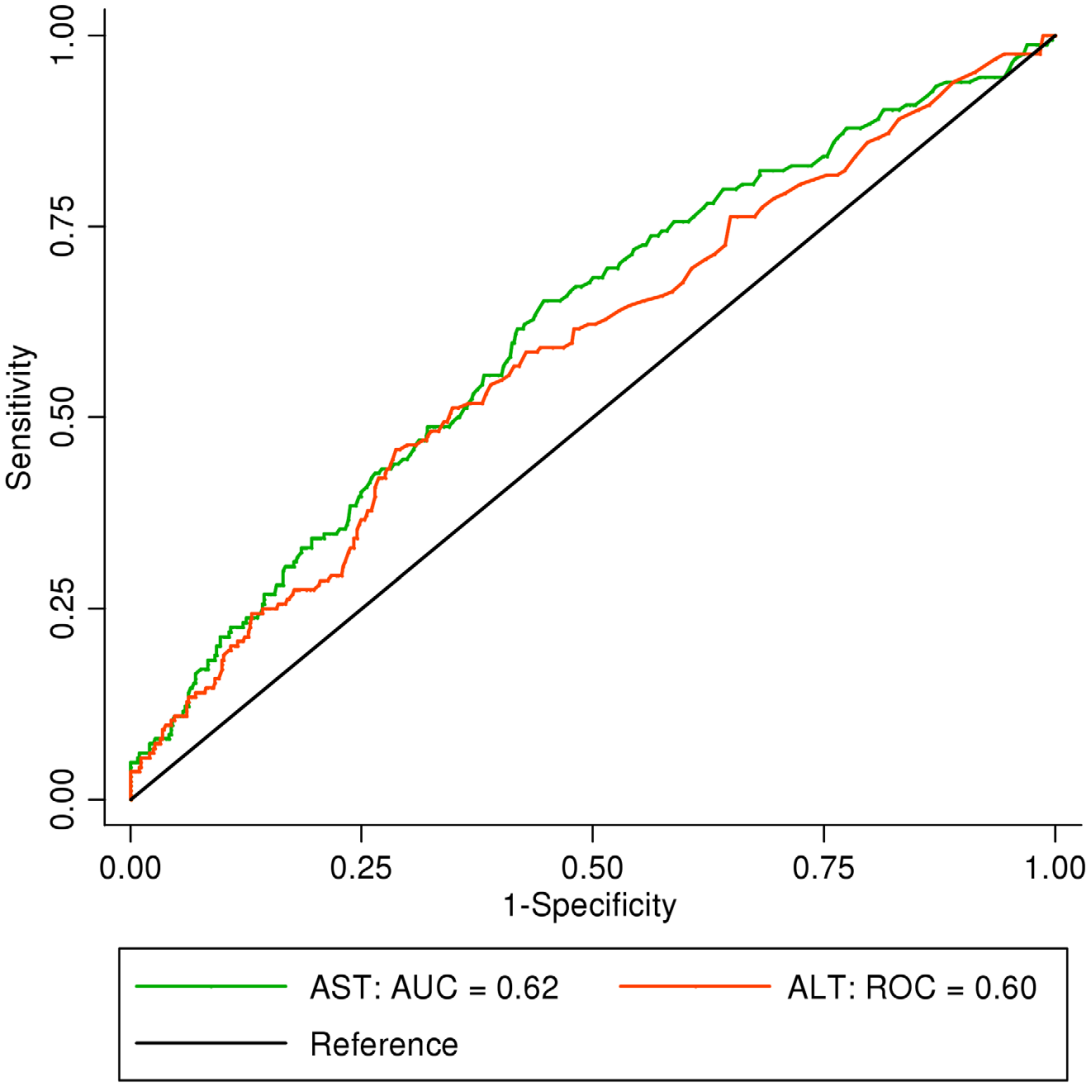
Discriminatory performance of AST and ALT for differentiating severe dengue from non-severe dengue. AST = aspartate aminotransferase. ALT = alanine aminotransferase. AUC = area under the curve.

**Figure 2:**
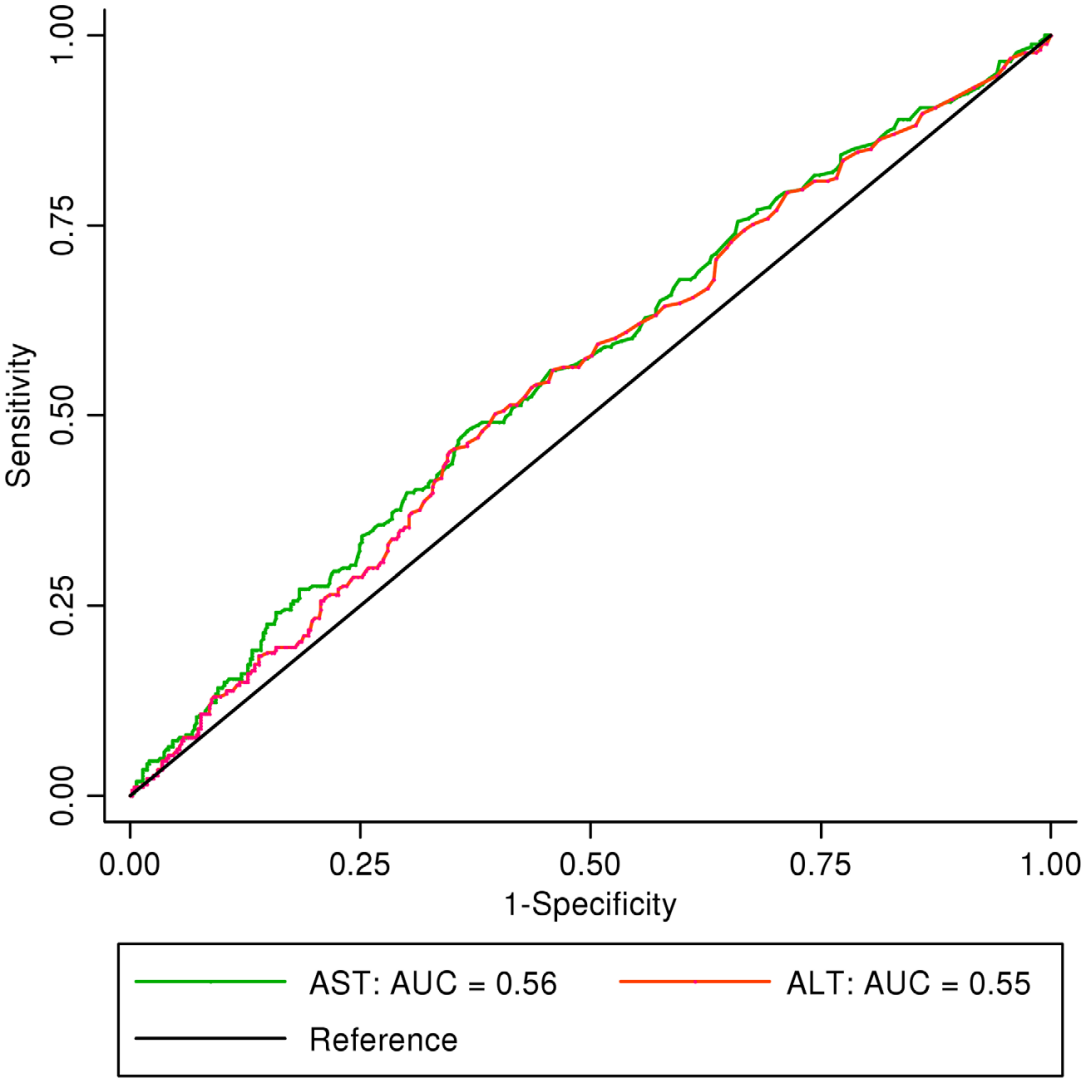
Discriminatory performance of AST and ALT for differentiating dengue hemorrhagic fever from dengue fever. AST = aspartate aminotransferase. ALT = alanine aminotransferase. AUC = area under the curve.

In order to determine the reliability of AST and ALT values in defining dengue severity, ROC curves for AST and ALT against severe dengue excluding isolated transaminitis were determined (Figure 1). The AUC for AST was 0.62 (95% confidence interval [CI]: 0.57–0.67) and for ALT, 0.60 (95% CI: 0.54–0.64). This demonstrates that AST or ALT levels are insufficient to differentiate among the WHO 2009 dengue classifications. They were also poorly discriminatory between DF and DHF, as the areas under the curve (AUC) for AST and ALT were 0.56 (95% CI: 0.52–0.61) and 0.55 (95% CI: 0.51–0.59), respectively (Figure 2). In our serology-positive cohort, the AUC values for AST and ALT were 0.56 and 0.54 for differentiating between DF and DHF. The AUC values for severe and non-severe dengue were 0.64 and 0.60 for AST and ALT, respectively.

**Figure 3:**
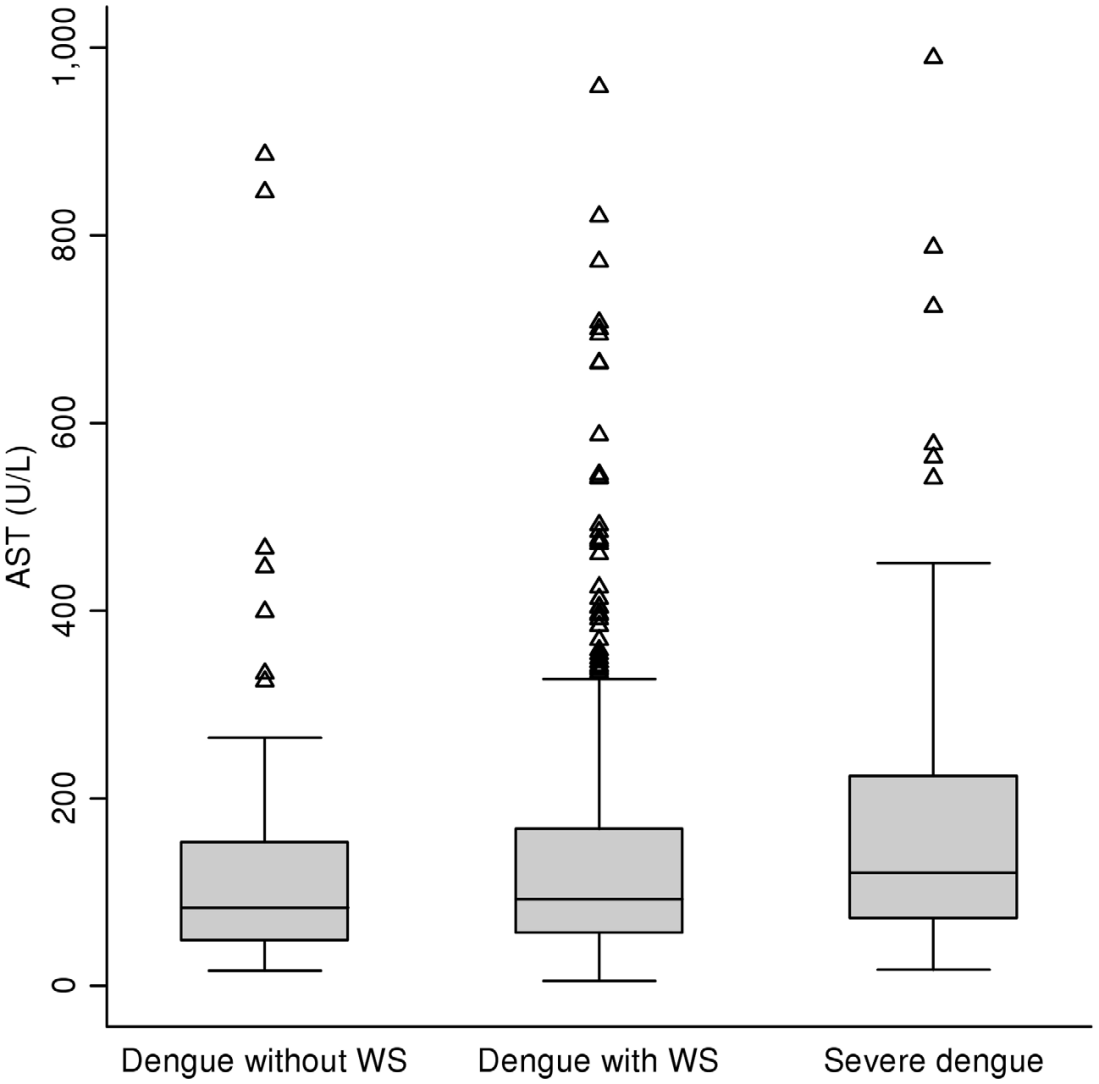
Distribution of maximum AST values during hospitalization for those with AST<1000 U/L: WHO 2009 classifications. The 25^th^ and 75^th^ percentiles are represented by the lower and upper horizontal edges of the box, respectively, while the whiskers represent the 5^th^ and 95^th^ percentiles. The median is indicated by the horizontal line inside the box. WS = warning signs. AST = aspartate aminotransferase. U/L = units/liter.

**Figure 4:**
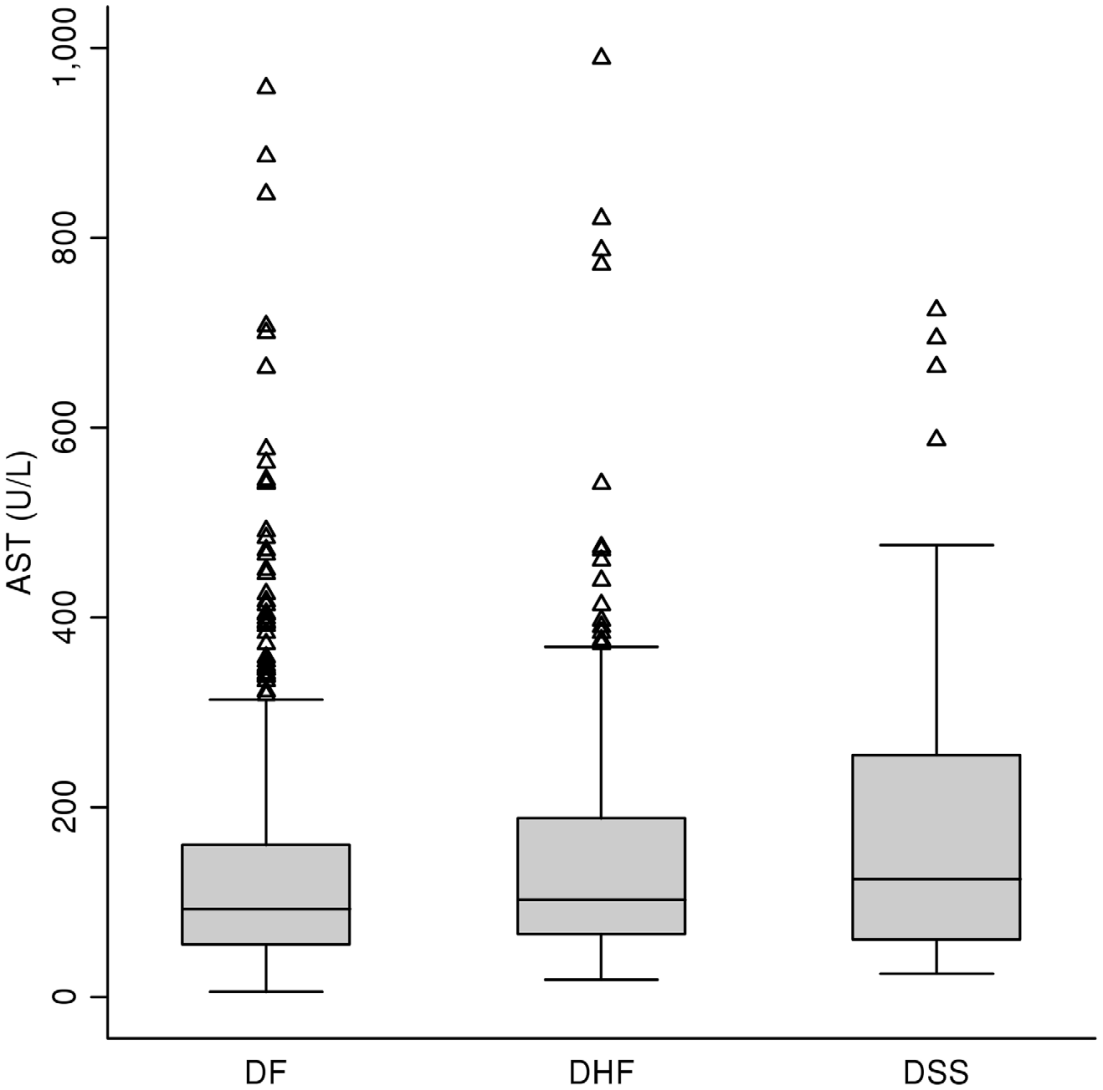
Distribution of maximum AST values during hospitalization for those with AST<1000 U/L: WHO 1997 classifications. AST = aspartate aminotransferase. U/L = units/liter. DF = dengue fever. DHF = dengue hemorrhagic fever. DSS = dengue shock syndrome.

The box plots in Figure 3 for the distributions of AST values show considerable overlap among the liver enzyme values for those with dengue with and without warning signs, and severe dengue. Because there were extreme outliers in our cohort, only those with AST below 1000 U/L were included in these plots. Figure 4 shows overlapping AST values among those with DF and DHF. Similarly, considerable overlap was observed in ALT values for patients with dengue with and without warning signs, and severe dengue, as well as for DF versus DHF (data not shown).

## Discussion

Our analysis showed that liver aminotransferase levels were associated with but did not adequately differentiate between dengue severity. Although median AST and ALT values were significantly higher in those with DHF/DSS versus DF, and severe dengue versus non-severe dengue, very few (1.0%) had AST or ALT≥1000 U/L. Notably, none developed liver failure, and death occurred in only 1 patient (0.1%). The majority of patients recovered uneventfully.

The lack of acute liver failure in our study was not unusual, as the incidence of acute liver failure in dengue patients was 1.1% in studies by Trung and Kuo [4], [6]. The largest study to date reported no acute fulminant hepatitis [3]. In contrast to these adult studies, it is noteworthy that in dengue-endemic countries, dengue may be an important cause of acute liver failure in children [21], [22].

While some studies have shown that AST and ALT values differ between DF and DHF [3], [4], [8], few studies support AST or ALT as an independent predictor of DHF [23]. Two studies in Singapore found liver aminotransferase levels to be significantly elevated among DF and DHF patients [12] and survivors and non-survivors of dengue [24] on univariate analysis, but this association was lost after adjusting for confounders on multivariate analysis.

Trung et al. showed significant differences comparing other febrile illness, dengue without plasma leakage, and dengue with plasma leakage with and without shock during critical and convalescent phases for AST but during critical phase for ALT only [4]. We made the novel finding that liver aminotransferase levels may significantly vary according to dengue severity during the febrile phase. For DHF by WHO 1997 classification, both AST and ALT were significantly higher during the febrile phase compared to DF or DSS, and for severe dengue by WHO 2009, AST and ALT were significantly higher during the febrile and critical phases.

The impact of co-infection with hepatitis viruses or concomitant hepatotoxic drugs was not assessed in our retrospective study, although we did exclude those with known liver comorbidities. Kuo et al. found that hepatitis B or C did not increase the extent of liver aminotransferase elevation in a retrospective adult dengue study in Taiwan [6]. In contrast, Trung et al. found that hepatitis B co-infection modestly increased ALT levels without significant clinical impact in a prospective adult dengue study in Vietnam [4]. Tang et al. showed that dengue and hepatitis B co-infected patients showed an aberrant cytokine secretion profile compared with those with dengue alone. [25]. In Singapore, seroprevalence for hepatitis B was 2.8% [26] and hepatitis C 0.37% [27].

The etiology of elevated aminotransferase levels during acute dengue illness is unclear since AST is expressed in the heart, skeletal muscle, red blood cells, kidneys, brain, and liver, while ALT is secreted primarily by the liver [28], [29]. Because dengue infection can cause acute damage to these non-hepatic tissue types that express AST, raised aminotransferase levels may not be entirely due to severe liver involvement. It is therefore possible that the patients with high AST levels were also more likely to be classified as severe dengue under the 2009 criteria due to the common pathways to non-hepatic tissue damage, even though there is no association with poorer outcome.

Our retrospective study has some limitations. Aspartate and alanine aminotransferase values were tracked according to clinical judgment rather than at regular intervals during illness. We did not have dengue serotype data for each patient, but in 2006, DENV-1 was predominant in Singapore with a switch to DENV-2 in 2007–2008 [30]. Serology-positive cases were not included in primary analyses because our clinical laboratory used a rapid diagnostic test with potential for false positive results [31], we did not have paired sera to confirm dengue diagnosis [9], and not every patient with elevated AST or ALT was comprehensively evaluated for other etiologies of viral and non-viral hepatitis. Although serology-positive cases presented later during illness, we saw no difference in outcome. Five serology-positive patients (0.34%) required ICU admission versus 0.43% of PCR-positive cases, while four patients (0.27%) died in the serology-positive cohort, versus 1 patient (0.14%) among PCR-positive cases. However, relative data accuracy in our retrospective study was made possible by using a standardized dengue clinical care path. Another limitation of this study is the relatively few cases with substantially elevated liver aminotransferase levels. At the same time, since our cohort comprised primarily adults, additional studies in pediatric populations will be useful to confirm our findings.

In patients with DHF/DSS or severe dengue, early diagnosis and proper management may improve outcome in most patients without comorbidities. However, in resource-limited countries, patients with severe disease may present late to the hospital with shock, with or without organ impairment at the time of admission. Our study highlights that early diagnosis and proper management of dengue patients may lead to excellent prognosis without organ injury.

In conclusion, elevated aminotransferase levels were associated with DHF/DSS and severe dengue in our cohort of adult patients with confirmed dengue. However, no threshold values discriminated between DF and DHF or between severe dengue and non-severe dengue.
